# Prediction of Histological Subtype Based on Segmental Localization of Malignant Pulmonary Nodule in the Upper Lobe of the Lung

**DOI:** 10.1111/1759-7714.70277

**Published:** 2026-03-30

**Authors:** Aysegul Gencer, Ersan Atahan, Bilun Gemicioglu, Sermin Borekci, Buket Caliskaner Ozturk

**Affiliations:** ^1^ Istanbul University‐Cerrahpasa, Cerrahpasa Faculty of Medicine Department of Pulmonary Diseases Istanbul Turkey

**Keywords:** lung cancer, lung cancer histological subtypes, malign nodule segmenter location, malignant pulmonary nodule

## Abstract

**Background:**

Studies have shown that malignant pulmonary nodules frequently occur in the upper lobes of the lung. However, there is no data on their distribution according to segments. The aim of this study is to predict the histological subtype of lung cancer based on the segment localization of malignant pulmonary nodules located in the upper lobe of the lung.

**Methods:**

Between March 1, 2018, and November 1, 2024, 2402 patients who applied to the pulmonary diseases outpatient clinic and were scheduled for further investigation after thoracic CT scans revealed pulmonary nodules measuring 8 mm or larger were screened. The demographic characteristics, pulmonary nodule size, and histological subtypes of 154 patients diagnosed with non‐small cell lung cancer (NSCLC) in further investigations were compared according to the upper lobe segments of the lung: apicoposterior and anterior.

**Results:**

Adenocarcinoma percentage was significantly higher in the apicoposterior group, while squamous cell carcinoma percentage was significantly higher in the anterior group (*p* = 0.010).

**Conclusions:**

NSCLC localized in the apicoposterior segment of the upper lobes of the lung tends to be adenocarcinoma, while those localized in the anterior segment tend to be squamous cell carcinoma. Tumor size less than 3 cm with apicoposterior localization is independently associated with the adenocarcinoma histological subtype.

## Graphical abstract

This study investigated the segmental distribution of histological subtypes in the upper lobes, the most common lobar location for NSCLC. The percentage of adenocarcinoma was found to be significantly higher in the apicoposterior group, while the percentage of squamous cell carcinoma was found to be significantly higher in the anterior group. The apicoposterior location was found to have a sensitivity value of 85% for predicting adenocarcinoma.

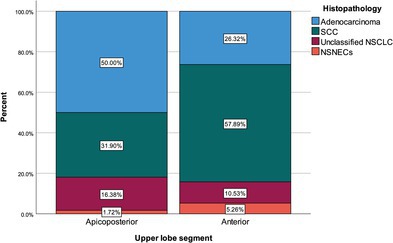

## 1 Introduction

Lung cancer is the leading cause of cancer‐related deaths worldwide [1]. According to 2020 global data, lung cancer is the second most commonly diagnosed cancer, causing 2.2 million new diagnoses and 1.8 million deaths annually [2]. Incidence and mortality rates are 3 to 4 times higher in developed countries compared to developing countries, but this is thought to change with the increasing tobacco epidemic in low‐income countries [3]. The highest incidence rates are in Micronesia/Polynesia, Eastern and Southern Europe, Eastern Asia, and Western Asia, while Turkey has the highest rate among men globally [3].

In high‐risk individuals, such as heavy smokers, screening with computed tomography (CT) is effective in the diagnosis of early‐stage lung cancer and treatment success becomes more likely [4, 5, 6, 7, 8]. Only 25%–30% of patients diagnosed with lung cancer present with localized disease. Five‐year survival is 73% in the early stage, while advanced disease has a poor prognosis with a survival rate of 13% [9, 10].

Considering that lung cancer can be detected at an early stage, the importance of pulmonary nodule screening with CT and nodule management protocols emerges [1]. Four basic pulmonary nodule screening programs and the relationship between size, density, shape‐edge characteristics, and localization with malignancy have been evaluated in nodule management [5, 6, 7, 8]. Compared to benign nodules, malignant nodules were larger, subsolid, had a shape without smooth margins, and were more frequently localized in the upper lobe [7, 8, 11]. While it has been proven by these studies that pulmonary nodules localized in the upper lobes of the lung are more likely to be malignant, there is no data on their distribution according to lung segments or histologic subtypes.

The aim of this study is to investigate the correlation between segmental localization and NSCLC histological subtypes located in the upper lobes of the lungs.

## 2 Methods

### 2.1 Study Design and Setting

**FIGURE 1:**
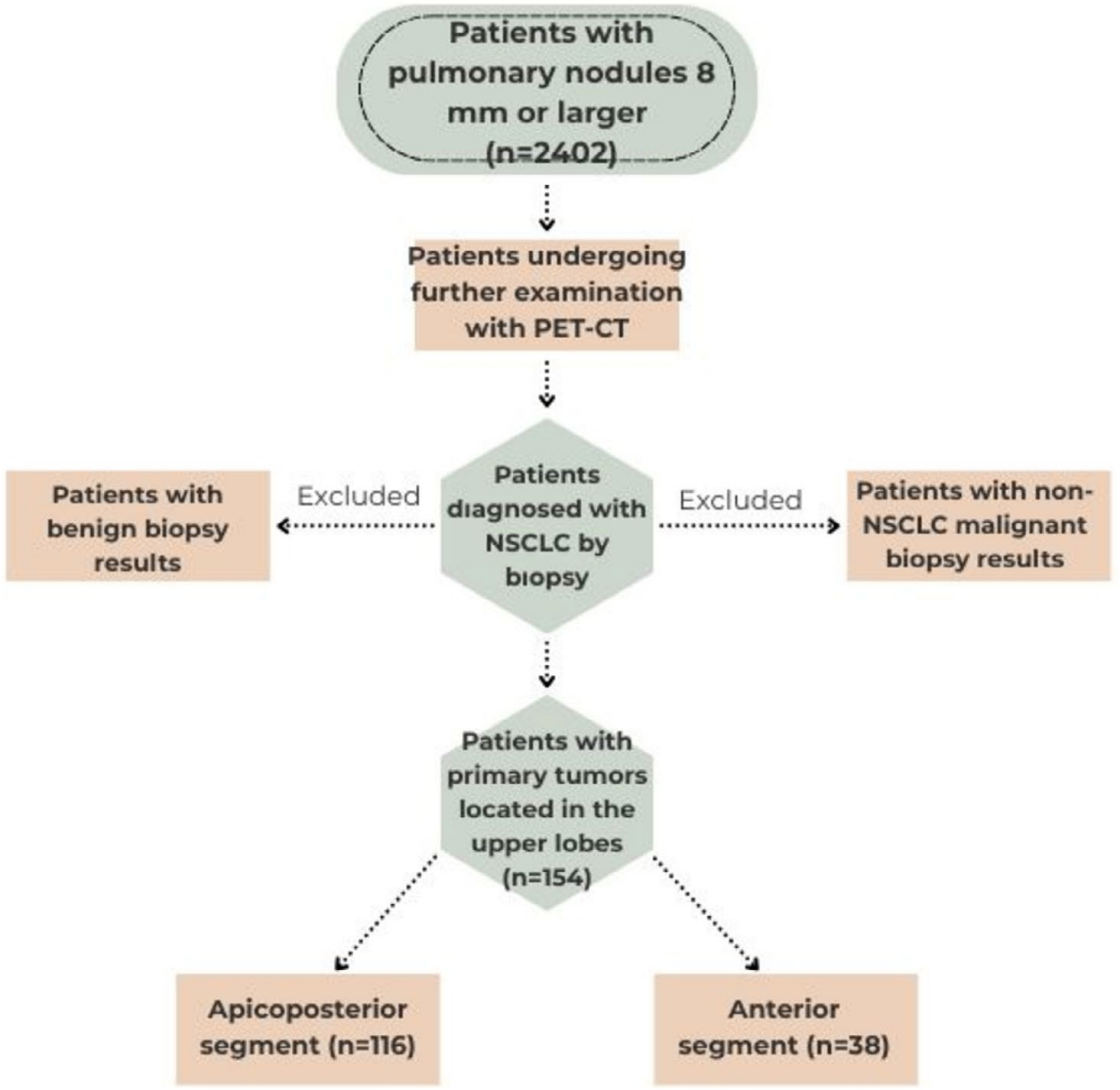
Flowchart of cohort selection.

This single‐center study, designed as a retrospective cohort, was conducted between March 1, 2018, and November 1, 2024. Data from 2402 patients who visited the Pulmonary Diseases outpatient clinic, had the largest pulmonary nodule measuring 8 mm or larger on chest CT scans, and were scheduled for further investigations for malignancy were analyzed. Following further examinations, data from 154 patients diagnosed with NSCLC were planned to be included in the study. The bilateral upper lung lobes were planned to be evaluated separately into two segments due to the difference between the right and left upper lobe segments: the apicoposterior segment and the anterior segment of the upper lobe (Figure 1).

This study was approved by the local clinical research ethics committee (no: E83045809‐604.01.01‐381 251). Written informed consent was obtained from all included patients.

### 2.2 Participants

#### 2.2.1 Inclusion Criteria

− Patients who underwent a thoracic CT scan at the pulmonary diseases outpatient clinic between March 1, 2018, and November 1, 2024, and had a pulmonary nodule of 8 mm or larger detected, with further investigation planned.
− Patients who underwent PET‐CT imaging.
− Patients diagnosed with NSCLC by biopsy.
− Patients with primary tumors located in the upper lobes.
− Patients aged 30–90 years.

#### 2.2.2 Exclusion Criteria

− Patients with benign biopsy results.
− Patients with malignancy other than non‐small cell lung cancer on biopsy (e.g., small cell lung cancer, metastases).
− Patients with a known history of lung cancer or any other malignancy
− Pregnancy
− Patients who do not provide written informed consent

### 2.3 Variables and Data Collection

Demographic data (age, gender), smoking status (pack‐years), and comorbidities were recorded from patient files. Pulmonary nodule characteristics were recorded from chest CT scans.

### 2.4 Thorax CT Scans

The data from the primary lesion identified as malignant in the upper lobes of the lung parenchymal windows on the thorax CT scan were included in the study. As the left upper lobe consists of two segments, the data from the right upper lobe were also added to the study as two segments: the apicoposterior segment and the anterior segment.

### 2.5 Diagnosis of Malignancy

Patients diagnosed with lung cancer by histopathological examination of biopsy samples were classified according to the World Health Organization classification of lung neoplasms [12].

### 2.6 Study Size

All patients who met all criteria and gave consent were included in the study.

### 2.7 Statistical Analysis

All analyses were conducted using IBM SPSS version 27 (IBM Corp., Armonk, NY, USA). A *p* value of less than 0.05 was considered statistically significant. Normality assumption was evaluated using histograms and Q‐Q plots. Descriptive statistics were presented using mean ± standard deviation for normally distributed continuous variables, median (25th percentile—75th percentile) for non‐normally distributed continuous variables, and frequency (percentage) for categorical variables. Between groups comparisons of continuous variables were performed using the Student's *t*‐test or Mann–Whitney U test depending on the normality assumption. Between groups comparisons of categorical variables were performed using the chi‐square test or Fisher's exact test or Fisher–Freeman–Halton test. Adenocarcinoma prediction performance of the apicoposterior segment was assessed using receiver operating characteristic (ROC) curve analysis. Logistic regression analyses were performed to determine significant factors independently associated with adenocarcinoma. All variables were analyzed using univariable logistic regression analysis. Variables with a *p* value of less than 0.10 (sex, smoking pack‐year, segment, tumor size) and age as a potential confounder were included in multivariable logistic regression analysis.

## 3 Results

We included 154 patients (30 females and 124 males) into the study; mean age was 67.99 ± 11.06. One hundred and thirty‐six (88.31%) patients had a smoking history, and the median smoking pack‐year was 40 (interquartile range 30–60). Seven (4.55%) patients had a tuberculosis history, 2 (1.30%) patients had asbestos exposure history, and 16 (10.39%) patients had lung cancer history in their family.

**FIGURE 2:**
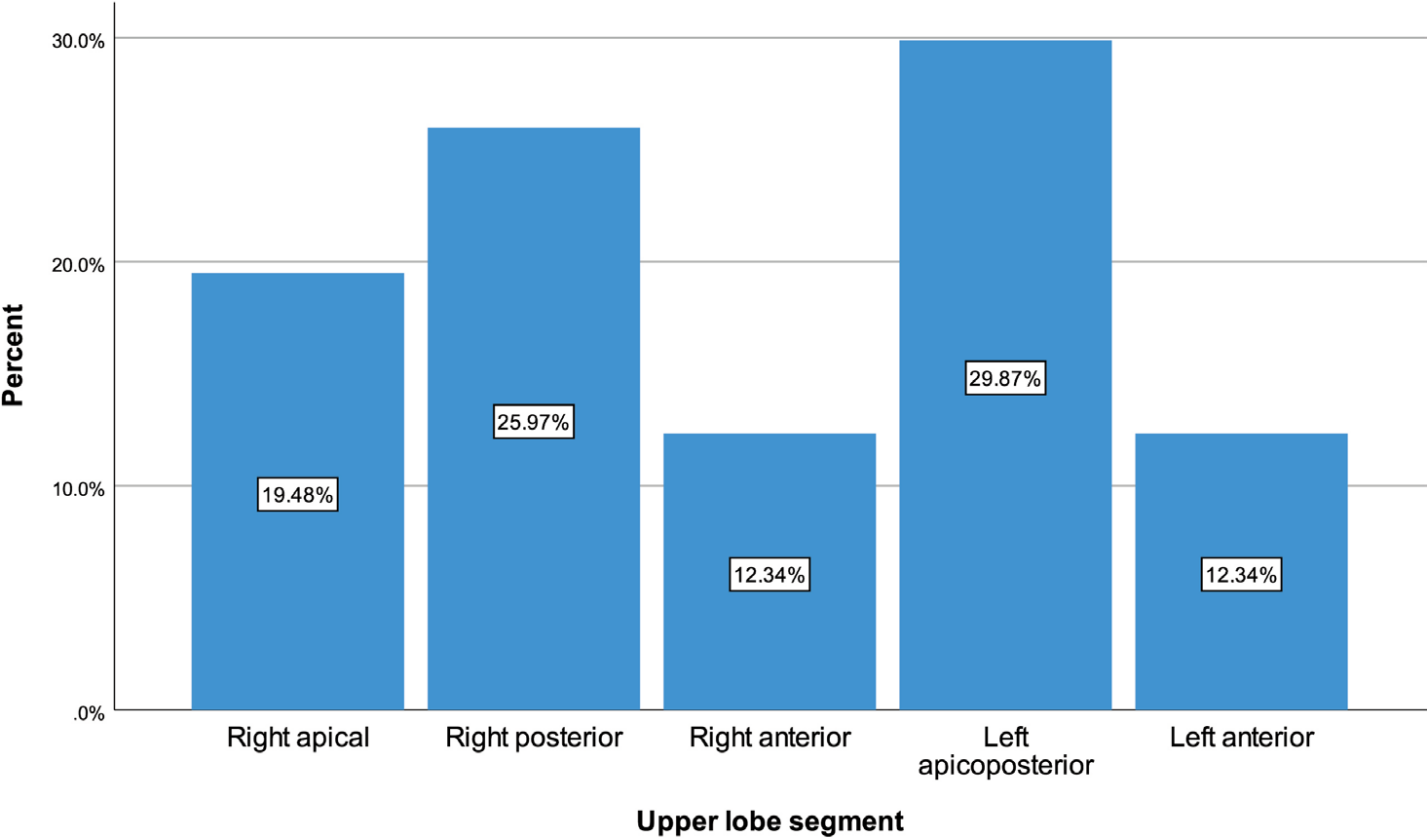
Distribution of upper lobe segments.

Thirty (19.48%) tumors were located in the right upper lobe apical segment, 40 (25.97%) in the right upper lobe posterior segment, 19 (12.34%) in the right upper lobe anterior segment, 46 (29.87%) in the left upper lobe apicoposterior segment, and 19 (12.34%) in the left upper lobe anterior segment. Median tumor size was 40 (interquartile range 23–63) mm, and 101 (65.58%) tumors were equal to or larger than 30 mm. Histopathology of the tumors was adenocarcinoma for 68 (44.16%) tumors, squamous cell carcinoma for 59 (38.31%) tumors, unclassified non‐small cell lung carcinoma for 23 (14.94%) tumors, and non‐small cell neuroendocrine carcinoma for 4 (2.60%) tumors (Table 1) (Figure 2).

**TABLE 1:** Summary of demographics and tumor characteristics.

Age	67.99 ± 11.06
Sex	
Female	30 (19.48%)
Male	124 (80.52%)
Smoking history	136 (88.31%)
Smoking pack‐year	40 (30–60)
Hypertension	30 (19.48%)
Coronary artery disease	22 (14.29%)
Diabetes mellitus	19 (12.34%)
COPD	25 (16.23%)
Tuberculosis history	7 (4.55%)
Asbestos exposure	2 (1.30%)
Malignancy history in family	21 (13.64%)
Lung cancer history in family	16 (10.39%)
Side	
Right	89 (57.79%)
Left	65 (42.21%)
Segment	
Right upper lobe apical	30 (19.48%)
Right upper lobe posterior	40 (25.97%)
Right upper lobe anterior	19 (12.34%)
Left upper lobe apicoposterior	46 (29.87%)
Left upper lobe anterior	19 (12.34%)
Tumor size, mm	40 (23–63)
< 30 mm	53 (34.42%)
≥ 30 mm	101 (65.58%)
Histopathology	
Adenocarcinoma	68 (44.16%)
SCC	59 (38.31%)
Unclassified NSCLC	23 (14.94%)
NSNECs	4 (2.60%)

*Note:* Descriptive statistics are presented using mean ± standard deviation for normally distributed continuous variables, median (25th percentile—75th percentile) for non‐normally distributed continuous variables and frequency (percentage) for categorical variables.

Abbreviations: COPD: chronic obstructive pulmonary disease; NSCLC: non‐small cell lung carcinoma; NSNECs: non‐small cell neuroendocrine carcinomas; SCC: squamous cell carcinoma.

**FIGURE 3:**
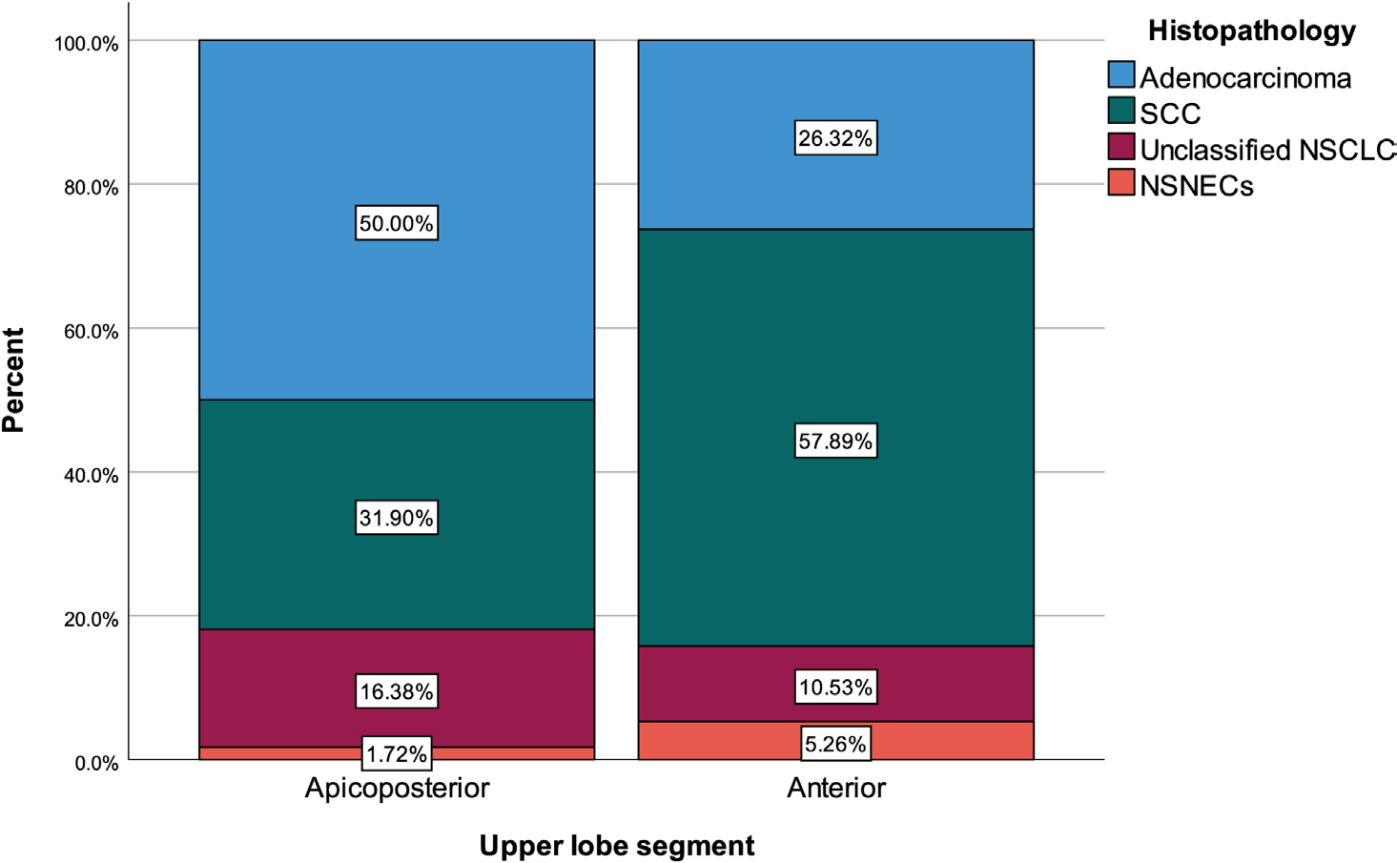
Histopathology distribution with regard to the upper lobe segment.

We divided patients into two groups according to the upper lobe segment as apicoposterior (116 patients) and anterior (38 patients). Adenocarcinoma percentage was significantly higher in the apicoposterior group, while squamous cell carcinoma percentage was significantly higher in the anterior group (*p* = 0.010). We found no significant differences between apicoposterior and anterior groups in terms of age, sex, smoking history, smoking pack‐year, comorbidities, tuberculosis history, asbestos exposure history, malignancy history in family, side and tumor size (Table 2) (Figure 3).

**TABLE 2:** Summary of demographics and tumor characteristics with regard to upper lobe segment.

	Upper lobe segment		*p*
	Apicoposterior (*n* = 116)	Anterior (*n* = 38)	
Age, years	67.85 ± 10.73	68.39 ± 12.16	0.794^†^
Sex			
Female	18 (15.52%)	12 (31.58%)	0.053^§^
Male	98 (84.48%)	26 (68.42%)	
Smoking history	103 (88.79%)	33 (86.84%)	0.773^#^
Smoking pack‐year	40 (30–60)	40 (20–50)	0.254^‡^
Hypertension	21 (18.10%)	9 (23.68%)	0.605^§^
Coronary artery disease	17 (14.66%)	5 (13.16%)	1.000^§^
Diabetes mellitus	14 (12.07%)	5 (13.16%)	1.000^#^
COPD	20 (17.24%)	5 (13.16%)	0.735^§^
Tuberculosis history	6 (5.17%)	1 (2.63%)	1.000^#^
Asbestos exposure	1 (0.86%)	1 (2.63%)	0.434^#^
Malignancy history in the family	16 (13.79%)	5 (13.16%)	1.000^§^
Lung cancer history in family	13 (11.21%)	3 (7.89%)	0.762^#^
Side			
Right	70 (60.34%)	19 (50.00%)	0.352^§^
Left	46 (39.66%)	19 (50.00%)	
Tumor size, mm	40 (24–65)	35.5 (22–60)	0.701^‡^
< 30 mm	37 (31.90%)	16 (42.11%)	0.341^§^
≥ 30 mm	79 (68.10%)	22 (57.89%)	
Histopathology			
Adenocarcinoma	58 (50.00%)	10 (26.32%)*	**0.010** ^¶^
SCC	37 (31.90%)	22 (57.89%)*	
Unclassified NSCLC	19 (16.38%)	4 (10.53%)	
NSNECs	2 (1.72%)	2 (5.26%)	

*Note:* Descriptive statistics are presented using mean ± standard deviation for normally distributed continuous variables, median (25th percentile–75th percentile) for non‐normally distributed continuous variables and frequency (percentage) for categorical variables.

Abbreviations: COPD: chronic obstructive pulmonary disease; NSCLC: non‐small cell lung carcinoma; NSNECs: non‐small cell neuroendocrine carcinomas; SCC: squamous cell carcinoma.

^†^ Student's *t*‐test.

^‡^ Mann Whitney *U* test.

^§^ Chi‐square test.

^#^ Fisher's exact test.

^¶^ Fisher–Freeman–Halton test.

* Statistically significant category for the variables with three or more categories. Statistically significant *p* values are shown in bold.

**FIGURE 4:**
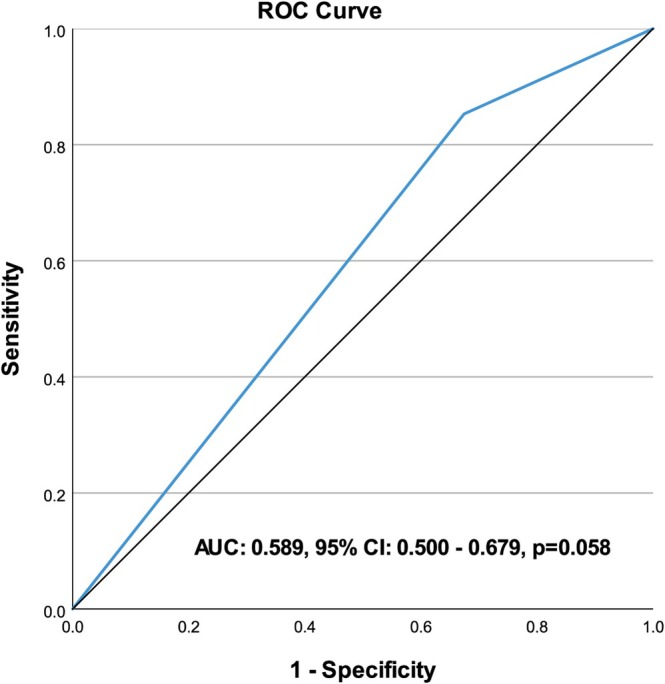
ROC curve of the apicoposterior segment to predict adenocarcinoma.

Apicoposterior location had 85.29% sensitivity, 32.56% specificity, 55.84% accuracy, 50.00% positive predictive value and 73.68% negative predictive value to predict adenocarcinoma. Area under ROC curve was 0.589 (95% CI: 0.500–0.679) and prediction performance was found to be nonsignificant (*p* = 0.058, Table 3) (Figure 4).

**TABLE 3:** Performance of apicoposterior segment to discriminate adenocarcinoma from other tumor types, ROC curve analysis.

Sensitivity	85.29%
Specificity	32.56%
Accuracy	55.84%
PPV	50.00%
NPV	73.68%
AUC (95% CI)	0.589 (0.500–0.679)
*p*	0.058

Abbreviations: AUC: area under ROC curve; CI: confidence interval; NPV: negative predictive value; PPV: positive predictive value; ROC: receiver operating characteristic.

**FIGURE 5:**
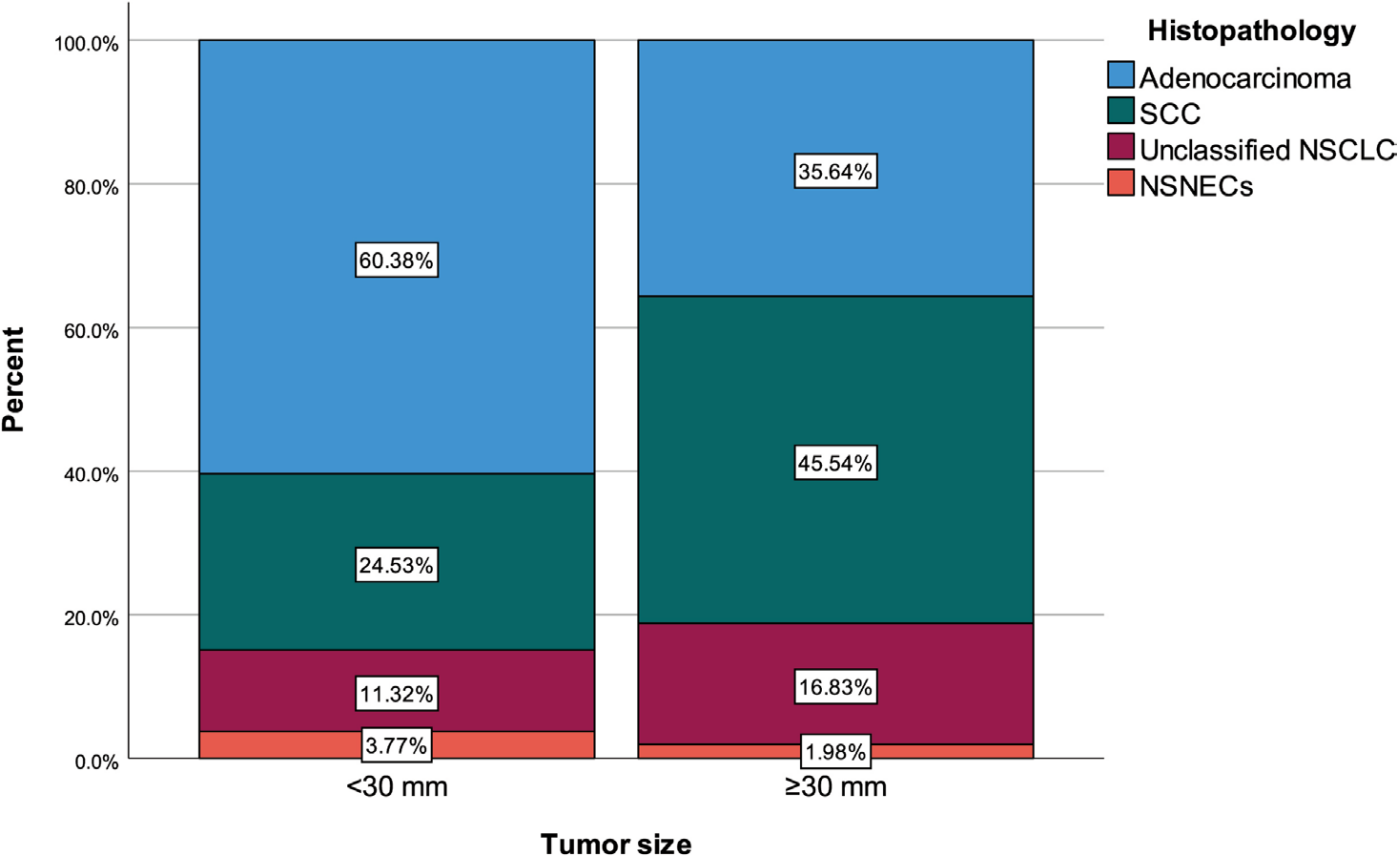
Histopathology distribution with regard to tumor size.

According to multivariable logistic regression analysis results, apicoposterior upper lobe segment (OR: 4.528, 95% CI: 1.808–11.342, *p* = 0.001) and low (< 30 mm) tumor size (OR: 3.283, 95% CI: 1.554–6.939, *p* = 0.002) were independently associated with adenocarcinoma after adjustment for age, sex, smoking pack‐year, and each other (Table 4) (Figure 5).

**TABLE 4:** Odds ratios for adenocarcinoma, logistic regression analysis results.

	Univariable		Multivariable	
	OR (95% CI)	*p*	OR (95% CI)	*p*
Age, years	1.002 (0.974–1.032)	0.873	1.003 (0.972–1.035)	0.841
Sex, Female	2.220 (0.984–5.009)	0.055	2.515 (0.927–6.823)	0.070
Smoking history, Yes	0.595 (0.221–1.601)	0.304		
Smoking pack‐year	0.991 (0.980–1.001)	0.088	0.993 (0.981–1.005)	0.266
Hypertension, Yes	0.810 (0.360–1.823)	0.610		
Coronary artery disease, Yes	0.686 (0.270–1.745)	0.428		
Diabetes mellitus, Yes	0.708 (0.262–1.908)	0.494		
COPD, Yes	0.992 (0.419–2.352)	0.986		
Tuberculosis history, Yes	0.946 (0.204–4.378)	0.944		
Asbestos exposure, Yes	1.269 (0.078–20.660)	0.867		
Malignancy history in family, Yes	1.176 (0.467–2.957)	0.731		
Lung cancer history in family, Yes	1.300 (0.461–3.664)	0.620		
Side, Left	0.599 (0.312–1.150)	0.124		
Segment, Apicoposterior	2.800 (1.247–6.285)	**0.013**	4.528 (1.808–11.342)	**0.001**
Tumor size, < 30 mm	2.751 (1.387–5.456)	**0.004**	3.283 (1.554–6.939)	**0.002**
Nagelkerke *R* ^2^	—		0.201	

*Note:* Statistically significant *p* values are shown in bold.

Abbreviations: CI: confidence interval; COPD: chronic obstructive pulmonary disease; OR: odds ratio.

## 4 Discussion

This study investigated the segmental distribution of histological subtypes in the upper lobes, which are the most common lobar location for NSCLC. The percentage of adenocarcinoma was found to be significantly higher in the apicoposterior group, while the percentage of squamous cell carcinoma was found to be significantly higher in the anterior group. Apicoposterior location was found to have a sensitivity value of 85% for predicting adenocarcinoma.

Studies on the locations of NSCLC, which accounts for 90% of all lung cancers, date back twenty‐five years. These studies indicated that squamous cells preferentially have a central (bronchial) localization, while adenocarcinomas frequently have a peripheral (bronchioloalveolar) localization [13]. In another study evaluating CT findings, squamous cell carcinoma showed a higher incidence in central locations, while adenocarcinoma tended to occur in peripheral locations [14]. Many studies in this direction have categorized the assessment according to localization in NSCLC as “central type” and “peripheral type” or “bronchus” and “non‐bronchus.” The assessment has not been evaluated based on segment localization to this extent [15].

In the Pan‐Canadian Lung Cancer Early Detection Study, it was shown that pulmonary nodules were frequently localized in the upper lobes of the lung [7]. Similar data were included in the review on lung cancer screening by Vlahos et al. and it was emphasized that pulmonary nodules were more commonly localized in the upper lobe [15, 16]. In the NELSON study, it was emphasized that malignant pulmonary nodules were frequently located in the upper lobes [8]. In line with these studies, our study included malignant nodules in the upper lobes diagnosed with NSCLC.

In the Pan‐Canadian Lung Cancer Early Detection Study, which also examined the distribution of malignant pulmonary nodules according to histological subtypes, adenocarcinoma was found to account for 73.5%–76.2% and squamous cell carcinoma for 11.9%–13.7% [7]. Data supporting this ranking in histological subtype classification were also found in our study in a similar order.

In the study by Deng et al., which evaluated 1842 patients, the following finding was made regarding segmental location: The apicoposterior segment of the upper lobe is the region where tumors are more frequently observed compared to the basal segments of the lower lobes, the epimeral segments of the lower lobes, the anterior segment of the upper lobes, and the middle lobe including the lingual lobe [17]. Our study also found that apicoposterior localization was more common, and this localization was found to be highly sensitive in terms of lung adenocarcinoma localization.

In the literature, there is very limited data showing the distribution of NSCLC diagnosis according to lung segments. In this respect, our study is pioneering in this field. Our data showed that tumors in the upper lobes of the lung tended to be located more frequently in the apicoposterior segment, with adenocarcinoma being the histological subtype most likely to be located in this area, with 85% sensitivity; it also showed that squamous cell carcinomas tended to be located more frequently in the anterior segment.

According to our data, in addition to the apicoposterior upper lobe segment, tumor size < 3 cm was independently associated with adenocarcinoma. A study by Wang et al. supports our data in terms of size comparison among NSCLC histological subtypes: the average tumor size in squamous cell carcinoma was found to be significantly larger than in adenocarcinoma. Tumor sizes were measured in radiological imaging, and the average size was determined to be 2.1 cm in adenocarcinomas and 4.3 cm in squamous cell carcinomas [18].

In our study, we found that adenocarcinoma more frequently occurred in the apicoposterior region of the upper lobe. The relatively poor perfusion of the upper lobes of the lung and slow lymphatic drainage along the peribronchial system have been interpreted by Gurney et al. as potentially leading to higher particle concentrations in this region and, consequently, a predisposition to lung disease [19]. This hypothesis may explain the predisposition of adenocarcinoma to the upper segments. Considering the tendency of effective pathologies such as pulmonary tuberculosis to affect the apicoposterior segment, a predisposition to adenocarcinoma may have developed in the sequelae regions [20].

A study on three‐dimensional particle accumulation models in bronchial lobar‐segmental airway bifurcations has revealed that some of the epithelial cells located on the carinal ridges can receive large doses that may be several hundred times higher than the average dose for the entire airway. This study strengthens the hypothesis that the distinct regional selectivity of neoplastic lesions may stem from increased accumulation of toxic particulate matter in bronchial airway branches [21]. In this context, the more frequent occurrence of squamous cell carcinoma in the anterior segment may be due to the anatomical opening of the anterior segment bronchus.

The limitations of this study include its modest sample size, which only included the upper lobes of the lungs, its single‐center nature, and its retrospective design.

The strengths of this study, to the best of our knowledge, are that it is the first of its kind. The upper lobe segments, which are the most common sites of lung cancer, were evaluated, and statistically significant results were obtained.

## 5 Conclusion

This study showed that NSCLC located in the upper lobes of the lung, localized in the apicoposterior segment, tends to be adenocarcinoma, while those localized in the anterior segment tends to be squamous cell carcinoma. An independent association between apicoposterior location and tumor size under 3 cm and adenocarcinoma was found.

## Author Contributions

**Aysegul Gencer:** conceptualization, investigation, writing – original draft, writing – review and editing, methodology, data curation, resources, software, formal analysis, validation, visualization, funding acquisition, project administration. **Ersan Atahan:** writing – review and editing, investigation. **Bilun Gemicioglu:** investigation, writing – review and editing, supervision. **Sermin Borekci:** supervision, writing – review and editing, investigation. **Buket Caliskaner Ozturk:** conceptualization, investigation, writing – review and editing, supervision.

## Funding

The authors have nothing to report.

## Ethics Statement

The study was approved by our Faculty Clinical Research Ethics Committee (no: E83045809‐604.01.01‐381 251).

## Conflicts of Interest

The authors declare no conflicts of interest.

## Data Availability

The data that support the findings of this study are available from the corresponding author upon reasonable request.
